# Frequencies of ALK rearrangements in lung adenocarcinoma subtypes: a study of 2299 Chinese cases

**DOI:** 10.1186/s40064-016-2607-5

**Published:** 2016-06-27

**Authors:** Yongfeng Yu, Zhengping Ding, Lei Zhu, Haohua Teng, Shun Lu

**Affiliations:** Shanghai Lung Cancer Center, Shanghai Chest Hospital, Shanghai Jiao Tong University, 241 West Huaihai Road, Xuhui District, Shanghai, 200030 China; Department of Pathology, Shanghai Chest Hospital, Shanghai Jiao Tong University, 241 West Huaihai Road, Xuhui District, Shanghai, 200030 China

**Keywords:** Lung adenocarcinoma, Histologic classification, ALK status

## Abstract

**Purpose:**

This study aimed to determine the relationship between ALK status and lung adenocarcinoma subtypes, according to the IALSC/ATS/ERS classification in Chinese patients.

**Methods:**

A reclassification of 2299 surgically resected lung adenocarcinomas was performed, and ALK status was detected by immunohistochemistry (Ventana Medical Systems) in Shanghai Chest Hospital.

**Results:**

ALK rearrangements were identified in 93 of 2299 tumors (4.0 %). The ALK rearrangements frequencies were: 14.8 % (16/108), 10.3 % (20/195), 7.6 % (13/170), 2.8 % (29/1035), 2.5 % (3/119), 2.0 % (11/539), 0.9 % (1/114), and 0 % (0/19) for variants of invasive adenocarcinoma, solid predominant, micropapillary predominant, acinar predominant, minimally invasive adenocarcinoma, papillary predominant, lepidic predominant, and adenocarcinoma in situ, respectively.

**Conclusions:**

We reported significant discrepancies of ALK status in lung adenocarcinoma subtypes in Chinese patients.

**Electronic supplementary material:**

The online version of this article (doi:10.1186/s40064-016-2607-5) contains supplementary material, which is available to authorized users.

## Background

Lung cancer is the leading cause of cancer related mortality in both men and women worldwide (Jemal et al. 2011). Adenocarcinoma has become the most common histologic type of non–small cell lung cancer (NSCLC), accounting for nearly 40 % of all lung cancer cases, and it is a heterogeneous tumor. In 2011, the International Association for the Study of Lung Cancer (IASLC), the American Thoracic Society (ATS), and the European Respiratory Society (ERS) proposed a new classification system for lung adenocarcinoma (Travis et al. 2011). The 2015 WHO classification of lung adenocarcinoma is consistent with the IALSC/ATS/ERS classification in resection specimens (Travis et al. 2015).

ALK rearrangements in NSCLC were first described in lung adenocarcinomas. Approximately 3–6 % of lung adenocarcinoma were shown to harbor rearrangements of the ALK gene, which has been demonstrated to be a potent oncogenic driver and a promising therapeutic target (Paik et al. 2011). The US Food and Drug Administration (FDA) has approved crizotinib to treat locally advanced or metastatic ALK rearrangements lung adenocarcinomas (Shaw et al. 2013). Detecting ALK rearrangement is emerging as an important component of the pathologic analysis of lung adenocarcinomas. However, whether ALK status in lung adenocarcinomas correlates with histologic subtypes remains unclear.

The aim of this study was to evaluate whether or not the proposed IASLC/ATS/ERS classification correlates with ALK status in Chinese patients.

## Methods

### Patients and eligibility

All patients had received curative surgery with pathologic stage I to stage III lung adenocarcinomas at Shanghai Chest Hospital between July 2013 and December 2014. These patients did not receive previous chemotherapy or radiotherapy before surgery. Histological typing confirmed the lung adenocarcinoma according to the 2004 World Health Organization classification criteria. Lung cancer staging was performed for all the patients according to the seventh TNM classification. For all patients, medical records were reviewed to extract data on clinicopathologic characteristics. This study was approved by Shanghai Chest Hospital Ethics Committee.

### Histological evaluation

All these tumor samples were fixed in 10 % neutral buffered formalin, embedded in paraffin and stained with hematoxylin and eosin in the routine manner. Each of the slides was reviewed by two pathologists independently. Any discrepancies between the pathologists during determination of predominant subtypes were resolved via consensus by using a multiple-headed microscope. The average number of slides from each case reviewed in the this study was 8 (range 4–26). According to the IASLC/ATS/ERS classification scheme, each tumor was examined using comprehensive histologic subtyping, recording the percentage in 5 % increments for each histologic component. Tumors were classified as adenocarcinomas in situ, minimally invasive adenocarcinomas, and invasive adenocarcinomas, which were divided into lepidic predominant, papillary predominant, acinar predominant, micropapillary predominant and solid predominant. Variants of invasive adenocarcinoma included invasive mucinous adenocarcinoma and others. The predominant pattern was defined as the pattern with the greatest percentage.

### ALK immunohistochemistry

IHC was performed for all cases on 5-μm thick FFPE sections with the D5F3 rabbit anti-human monoclonal antibody (Cell Signaling Technologies) in a Bechmark XT staining module (Ventana Medical Systems, Illkirch Graffenstaden, France). The slides were dried at 60 °C for 1 h, deparaffinized using EZ Prep at 75 °C for 4 min, and incubated with the primary mAb at a dilution of 1:100 for 1 h at 37 °C for all samples. Detection was performed with the OptiView DAB IHC Detection Kit with signal amplification (Ventana Medical Systems). A positive external control consisting of a slide of a previously FISH-validated ALK-rearranged and IHC-positive sample was included. Negative controls consisted of the omission of the primary antibody and incubation with immunoglobulins of the same species. All sections were independently evaluated by two pathologists using a semi-quantitative system based on the H-index: 3× percentage of strongly staining cells + 2× percentage of moderately staining cells + percentage of weakly staining cells, giving “composite scores” that ranged from 0 to 300. Cases with the scores of 0 to 100 were interpreted as negative, 101 to 300 as positive.

### EGFR mutation analysis

Tumor samples were obtained from resected lesions. Tumor DNA was extracted using the QIAamp DNA FFPE tissue kit (Qiagen, Crawley, UK), and EGFR mutation was analyzed using the amplification refractory mutation system (ARMS)-based EGFR mutation detection kit (EGFR RGQ PCR kit, Qiagen, Crawley, UK) as instructed by the manufacturer. The commercial kit allows detection of 29 mutations in the EGFR gene.

### Statistical analysis

The Chi squared test was used to evaluate the relationships between ALK status and clinicopathologic variables. A probability level of 0.05 was chosen for statistical significance. Statistical analysis was performed with the SPSS 16 software (SPSS Inc, Chicago, Illinois, USA).

## Results

### Patient clinicopathologic characteristics

Of the 2299 lung adenocarcinoma patients, the median age was 60 years in the current cohort. There were 975 men and 1324 women. 1580 were never smokers and 719 were former or current smokers. Pathologic stages were stage I in 1866 patients, stage II in 189 patients, and stage III in 244 patients. According to the IALSC/ATS/ERS classification, the most prevalent subtype was acinar predominant (45.0 %), followed by papillary predominant (23.4 %), solid predominant (8.5 %), micropapillary predominant (7.4 %), minimally invasive adenocarcinoma (5.2 %), lepidic predominant (5.0 %), variants of invasive adenocarcinoma (4.7 %), and adenocarcinoma in situ (0.8 %). The clinicopathologic characteristics of the patients are listed in Table 1.

**Table 1:** Patient characteristics

Characteristics	Number (%)
Gender	
Male	975 (42.4)
Female	1324 (57.6)
Age (years)	
Range	21–83
Median	60
<65	1586 (69.0)
≥65	713 (31.0)
Smoking status	
Never	1580 (68.7)
Former/current	719 (31.3)
Stage	
I	1866 (81.2)
II	189 (8.2)
III	244 (10.6)
ALK status	
Positive	93 (4.0)
Negative	2206 (96.0)
Adenocarcinoma subtypes(IASLC/ATS/ERS)	
Adenocarcinoma in situ	19 (0.8)
Minimally invasive adenocarcinoma	119 (5.2)
Invasive adenocarcinoma	
Lepidic predominant	114 (5.0)
Acinar predominant	1035 (45.0)
Papillary predominant	539 (23.4)
Micropapillary predominant	170 (7.4)
Solid predominant	195 (8.5)
Variants of invasive adenocarcinoma	108 (4.7)

### Relationship between clinicopathologic characteristics and ALK status

Of the 2299 cases, ALK rearrangements were detected in 93 cases (4.0 %). ALK rearrangements were detected more frequently in younger age patients than in older age patients (5.0 vs. 1.8 %; p = 0.0003). ALK rearrangements were more frequent in patients with advanced clinical stage than in patients with early pathologic stage (6.7 % vs. 3.4 %; p = 0.002). Neither gender nor smoking status showed any significant association with frequencies of ALK rearrangements. Relationship between clinicopathological characteristics and ALK status is listed in Table 2. Among 93 cases with ALK rearrangements, 87 cases received EGFR mutation analysis. The overall frequency of concomitant EGFR mutations and ALK rearrangements were found in 6.9 % (6/87) ALK-rearranged patients.

**Table 2:** Relationship between clinicopathological characteristics and ALK status

	ALK positive number	ALK negative number	p
Total	93	2206	
Gender			
Male	38	937	0.76
Female	55	1269	
Age, (years)			
<65	80	1506	0.0003
≥65	13	700	
Smoking status			
Never	61	1519	0.51
Former/current	32	687	
Stage			
I	64	1802	0.002
II/III	29	404	

### Relationship between adenocarcinoma subtypes (IASLC/ATS/ERS) and ALK status

**Fig. 1:**
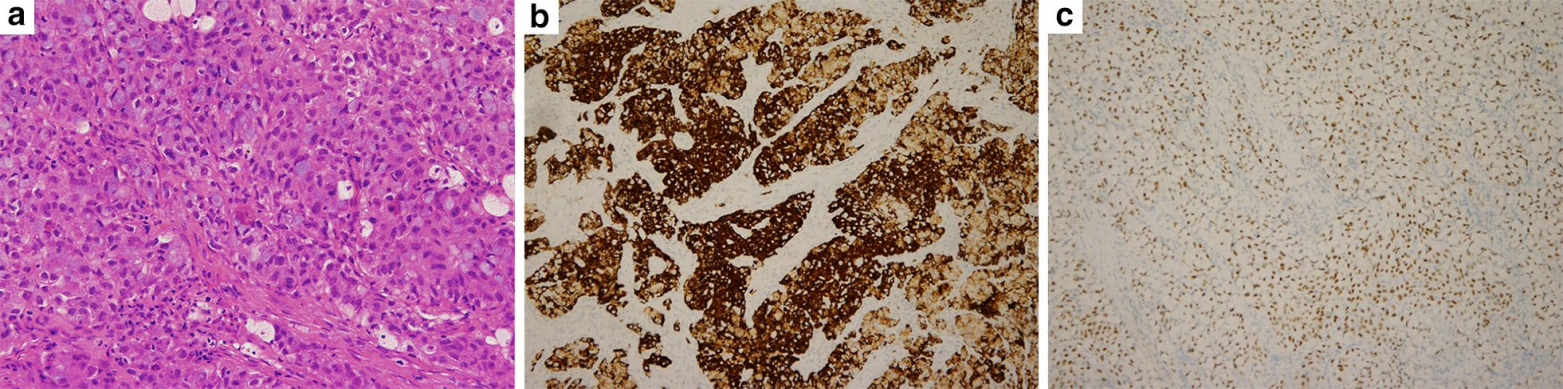
Detection of ALK rearrangements in lung adenocarcinoma patient (case No.253) by immunohistochemistry (IHC).** a** Solid predominant subtype of lung adenocarcinoma was shown by HE staining. Original magnification ×200. **b** Ventana IHC assay revealed strong expression of ALK in the patient. Original magnification ×200. **c** IHC result showed TTF-1 positive in the patient. Original magnification ×100

The ALK rearrangements frequencies were: 14.8 % (16/108) for variants of invasive adenocarcinoma (15 invasive mucinous adenocarcinoma and 1 colloid adenocarcinoma), 10.3 % (20/195) for solid predominant, 7.6 % (13/170) for micropapillary predominant, 2.8 % (29/1035) for acinar predominant, 2.5 % (3/119) for minimally invasive adenocarcinoma, 2.0 % (11/539) for papillary predominant, 0.9 % (1/114) for lepidic predominant, and 0 % (0/19) for adenocarcinoma in situ, respectively. ALK rearrangements were significantly associated with variants of invasive adenocarcinoma (p < 0.0001), solid predominant subtype (p < 0.0001), and micropapillary predominant subtype (p = 0.013). The relationship between adenocarcinoma subtypes (IASLC/ATS/ERS) and ALK status is shown in Table 3. Detection of ALK rearrangements in lung adenocarcinoma patient by immunohistochemistry (IHC) is shown in Fig. 1. Details on the clinicopathologic characteristics of the patients with ALK rearrangements are provided in Additional file 1: Table S1.

**Table 3:** Relationship between adenocarcinoma subtypes (IASLC/ATS/ERS) and ALK status

	ALK positive number	ALK negative number	P
Adenocarcinoma in situ			0.75
Yes	0 (0.0 %)	19	
No	93	2187	
Minimally invasive adenocarcinoma			0.53
Yes	3 (2.5 %)	116	
No	90	2090	
Lepidic predominant			0.13
Yes	1 (0.9 %)	113	
No	92	2093	
Acinar predominant			0.006
Yes	29 (2.8 %)	1006	
No	64	1200	
Papillary predominant			0.007
Yes	11(2.0 %)	528	
No	82	1678	
Micropapillary predominant			0.013
Yes	13 (7.6 %)	157	
No	80	2049	
Solid predominant			<0.0001
Yes	20 (10.3 %)	175	
No	73	2031	
Variants of invasive adenocarcinoma			<0.0001
Yes	16 (14.8 %)	92	
No	77	2114

## Discussion

ALK rearrangements were more often found in younger age patients, in never or light ex-smokers and in lung adenocarcinomas (Paik et al. 2011). Rodig et al. identified 20 (5.6 %) ALK rearrangements patients in 358 lung adenocarcinomas and found ALK rearrangements to be associated with younger age (p = 0.0002), never smoking (p < 0.0001) (Rodig et al. 2009). However, some previous studies found that the ALK rearrangements were not associated with smoking status (Koh et al. 2011). In our study, a total of 2299 consecutive and unselected patients who underwent definitive surgery for lung adenocarcinoma at the Shanghai Chest Hospital were reviewed retrospectively. We found ALK rearrangements was detected more frequently in younger age patients than in older age patients (5.0 vs. 1.8 %; p = 0.0003). In our cohort, 1580 cases (68.7 %) were never smokers. Meanwhile, there were a high proportion of female patients (1324 cases, 57.6 %). Because of cultural reasons, the majority of female Chinese patients were never smokers. The previous reports have showed ALK Rearrangement occurs most likely in never or light smokers. However, smoking status showed no significant association with frequencies of ALK rearrangements in our study (p = 0.51). We do not know the real reasons. On the other hand, the majority of patients had stage I tumors (1866 cases, 81.2 %). With low dose spiral CT scan widely used in physical examination, increasing number of patients with early-stage disease are detected in Shanghai, especially for the female patients, most of whom are never smokers. The relevant data is shown in the reports of Shanghai Municipal Center for Disease Control and Prevention (http://www.scdc.sh.cn). The reasons why the female population suffers lung cancer are still unknown. Kim et al. investigated 80 ALK-rearranged and 213 ALK-negative resected lung adenocarcinomas and observed that ALK-rearranged tumors were associated with frequent nodal metastasis, and higher stage of disease at diagnosis (Kim et al. 2014). Other previous studies also confirmed this results (Rodig et al. 2009; Blackhall et al. 2014). Our study showed that ALK rearrangements were more frequent in patients with advanced clinical stage than in patients with early pathologic stage (p = 0.002).

ALK-rearranged lung adenocarcinomas were associated with histologic subtypes. Nishino et al. analyzed specimens from 104 ALK rearrangements and 215 ALK negative lung adenocarcinomas and found that the majority (54 %) of ALK rearrangements tumors with signet ring cells demonstrated a solid predominant pattern (Nishino et al. 2012). In other studies, it was also reported that a solid histology with signet-ring cells was significantly associated with ALK rearrangements lung adenocarcinomas (Pareja et al. 2015; Pan et al. 2014). In our study, we found that ALK rearrangements frequencies were significantly higher in variants of invasive adenocarcinoma (14.8 %), solid predominant subtype (10.3 %), and micropapillary predominant subtype (7.6 %) than that of other subtypes. Up to now, our report is the largest Asian data in this field. The results of this study can therefore be more confidently generalized to the Chinese population than other publications.

In our study, the overall frequency of concomitant EGFR mutations and ALK rearrangements were found in 6.9 % (6/87) ALK rearranged patients. ALK rearrangements are often mutually exclusive with other oncogenic alterations detected in NSCLC including EGFR mutations. Yang et al. reported that the overall frequency of concomitant EGFR mutations and ALK rearrangements was 1.3 % (13/977). EGFR/ALK co-alterations were found in 3.9 % (13/336) EGFR-mutant and 18.6 % (13/70) ALK-rearranged patients (Yang et al. 2014). Sasaki et al. identified a subset (3/50; 6 %) of treatment naive NSCLC patients with ALK rearrangements that also had concurrent EGFR activating mutations. They further found the resistance mechanisms to ALK TKIs mediated by both ALK and by a bypass signaling pathway mediated by EGFR, and these mechanisms can occur independently, or in the same cancer (Sasaki et al. 2011).

Break-apart fluorescent in situ hybridization (FISH) is currently the only diagnostic tool approved by the FDA. The low incidence of ALK rearrangement (about 4 % in NSCLC) requires a more rapid and cost-efficient method for screening. Many countries have adopted immunohistochemistry (IHC) screening followed by FISH confirmation. In 2013, the IHC (Ventana Medical Systems) for ALK status was approved by China Food and Drug Administration (CFDA). A high concordance between IHC and FISH results has been reported. However, recent large-scaled studies have also found more than a few cases with discordant results between IHC and FISH. A single assay strategy can lead to inadequate selection of patients (Martin et al. 2015; Ilie et al. 2015). Some possible causes have been proposed. Intracellular and extracellular mucin can cause false-negative and false-positive results, respectively, in IHC analysis. In terms of FISH, atypical FISH signals, such as a 3′-5′-3′ red doublet pattern, and compressed z-stacked images for vertically split signals may give false-negative results. The unique method of IHC (Ventana Medical Systems) was the limitation of the present study.

In conclusion, we reported significant discrepancies of ALK status in lung adenocarcinoma subtypes according to the IALSC/ATS/ERS classification in Chinese patients.

## Additional file

10.1186/s40064-016-2607-5 Clinicopathologic characteristics of the patients with ALK rearrangements.
