# Supplementary material for: Frequencies of ALK rearrangements in lung adenocarcinoma subtypes: a study of 2299 Chinese cases
Source: Springerplus. 2016 Jun 27;5(1):894. doi: 10.1186/s40064-016-2607-5 (PMC4923004; doi:10.1186/s40064-016-2607-5)
Supplement: Supplementary file 1 — 10.1186/s40064-016-2607-5 Clinicopathologic characteristics of the patients with ALK rearrangements. [file 40064_2016_2607_MOESM1_ESM.doc]

**TABLE S1.** Clinicopathologic Characteristics of the Patients with ALK Rearrangements

| Case No. | Gender | Age(yr) | Smoking Status | Stage | Histopathologic Subtypes | ALK  IHC | TTF1  IHC | p63  IHC | EGFR mutation | FISH test  for ALK |
| --- | --- | --- | --- | --- | --- | --- | --- | --- | --- | --- |
| 40 | M | 58 | C | III | Papillary predominant | + | + | / | WT | / |
| 43 | M | 39 | N | I | Papillary predominant | + | + | - | WT | / |
| 58 | M | 57 | C | I | Solid predominant | + | + | / | / | / |
| 64 | M | 56 | C | I | Papillary predominant | + | + | / | WT | / |
| 78 | M | 49 | N | I | Solid predominant | + | + | - | WT | / |
| 79 | F | 58 | N | I | Papillary predominant | + | + | + | WT | / |
| 82 | M | 81 | C | I | Acinar predominant | + | + | / | WT | / |
| 84 | F | 54 | N | I | Solid predominant | W+ | + | - | WT | + |
| 156 | M | 71 | Former | I | Acinar predominant | + | + | / | / | / |
| 171 | F | 51 | N | I | Invasive mucinous adenocarcinoma | + | + | - | WT | / |
| 189 | F | 51 | N | I | Invasive mucinous adenocarcinoma | W+ | + | - | WT | + |
| 195 | M | 64 | C | I | Acinar predominant | + | + | - | WT | / |
| 198 | F | 40 | C | I | Acinar predominant | + | + | - | L858R | / |
| 242 | F | 56 | N | I | Acinar predominant | + | + | - | WT | / |
| 253 | M | 52 | C | I | Solid predominant | + | + | - | WT | / |
| 277 | M | 69 | N | I | Invasive mucinous adenocarcinoma | W+ | + | - | WT | + |
| 286 | M | 53 | Former | I | Papillary predominant | W+ | + | / | L858R | **-** |
| 291 | F | 59 | N | I | Acinar predominant | + | + | / | WT | / |
| 337 | F | 49 | N | I | Solid predominant | + | + | + | WT | / |
| 394 | F | 53 | N | I | Minimally invasive adenocarcinoma | + | + | - | WT | / |
| 395 | M | 53 | C | I | Solid predominant | + | + | - | WT | / |
| 441 | F | 65 | N | I | Solid predominant | + | + | - | WT | / |
| 475 | M | 34 | C | I | Acinar predominant | + | + | - | WT | / |
| 478 | F | 61 | N | I | Acinar predominant | + | + | - | WT | / |
| 490 | M | 48 | C | III | Solid predominant | + | + | - | WT | / |
| 521 | F | 42 | N | I | Solid predominant | W+ | + | / | WT | + |
| 598 | M | 49 | C | I | Acinar predominant | + | - | - | WT | / |
| 624 | F | 67 | N | II | Solid predominant | + | + | - | WT | / |
| 630 | F | 49 | N | I | Papillary predominant | + | + | - | L858R | / |
| 644 | F | 44 | N | III | Solid predominant | + | - | - | WT | / |
| 673 | M | 60 | C | III | Invasive mucinous adenocarcinoma | + | + | - | WT | / |
| 703 | F | 56 | N | I | Invasive mucinous adenocarcinoma | + | + | - | WT | / |
| 737 | F | 61 | N | II | Micropapillary predominant | W+ | + | - | WT | + |
| 760 | F | 48 | N | I | Acinar predominant | + | + | - | WT | / |
| 770 | F | 41 | N | II | Acinar predominant | + | + | - | / | / |
| 783 | F | 43 | N | I | Papillary predominant | + | + | - | WT | / |
| 819 | F | 52 | N | I | Invasive mucinous adenocarcinoma | + | + | - | WT | / |
| 838 | F | 61 | N | II | Micropapillary predominant | + | + | - | WT | / |
| 855 | F | 59 | N | I | Acinar predominant | + | + | - | WT | / |
| 875 | M | 56 | Former | I | Micropapillary predominant | + | + | - | WT | / |
| 878 | M | 50 | C | I | Solid predominant | W+ | + | - | WT | + |
| 971 | M | 47 | N | I | Micropapillary predominant | + | + | - | WT | / |
| 1001 | F | 34 | C | III | Colloid adenocarcinoma | + | + | - | WT | / |
| 1020 | F | 61 | Former | III | Acinar predominant | + | + | - | WT | / |
| 1048 | F | 51 | N | III | Acinar predominant | + | + | - | WT | / |
| 1076 | F | 50 | N | II | Invasive mucinous adenocarcinoma | + | + | - | WT | / |
| 1100 | M | 60 | C | I | Papillary predominant | + | + | - | WT | / |
| 1224 | F | 61 | N | I | Acinar predominant | + | + | - | WT | / |
| 1269 | F | 49 | N | I | Invasive mucinous adenocarcinoma | W+ | + | - | WT | - |
| 1274 | F | 44 | N | I | Acinar predominant | + | + | - | WT | / |
| 1330 | M | 46 | C | I | Micropapillary predominant | + | + | - | / | / |
| 1340 | F | 48 | N | III | Micropapillary predominant | + | + | - | WT | / |
| 1363 | M | 44 | C | III | Acinar predominant | + | + | - | WT | / |
| 1381 | F | 43 | N | III | Invasive mucinous adenocarcinoma | + | + | - | WT | / |
| 1458 | F | 55 | N | II | Micropapillary predominant | + | + | - | WT | / |
| 1462 | M | 68 | N | III | Micropapillary predominant | + | + | - | WT | / |
| 1503 | M | 60 | C | I | Minimally invasive adenocarcinoma | + | + | - | WT | / |
| 1518 | F | 36 | N | I | Acinar predominant | + | + | - | 19DEL | / |
| 1539 | M | 52 | C | III | Micropapillary predominant | + | + | - | / | / |
| 1558 | F | 45 | N | I | Acinar predominant | + | + | - | WT | / |
| 1608 | F | 27 | N | II | Papillary predominant | W+ | + | - | WT | + |
| 1612 | F | 57 | N | III | Solid predominant | + | + | - | WT | / |
| 1618 | M | 62 | C | I | Acinar predominant | + | + | - | WT | / |
| 1626 | F | 57 | N | I | Micropapillary predominant | + | + | - | WT | / |
| 1666 | M | 34 | C | III | Acinar predominant | W+ | + | - | WT | + |
| 1674 | M | 40 | N | I | Acinar predominant | + | + | + | WT | / |
| 1678 | F | 42 | N | I | Solid predominant | + | + | - | WT | / |
| 1687 | F | 44 | N | II | Acinar predominant | + | + | - | WT | / |
| 1699 | M | 45 | C | II | Acinar predominant | + | + | - | WT | / |
| 1704 | M | 46 | C | I | Micropapillary predominant | + | + | - | WT | / |
| 1719 | F | 48 | N | II | Acinar predominant | + | + | - | WT | / |
| 1720 | F | 48 | N | I | Acinar predominant | + | + | - | WT | / |
| 1723 | F | 49 | N | I | Invasive mucinous adenocarcinoma | + | + | - | WT | / |
| 1725 | F | 49 | N | I | Invasive mucinous adenocarcinoma | + | + | - | WT | / |
| 1727 | F | 49 | N | I | Acinar predominant | + | + | + | WT | / |
| 1762 | M | 52 | C | I | Minimally invasive adenocarcinoma | W+ | + | / | WT | + |
| 1766 | F | 52 | N | I | Acinar predominant | + | + | - | WT | / |
| 1778 | F | 53 | N | I | Micropapillary predominant | + | + | - | 19DEL | / |
| 1802 | F | 54 | N | I | Invasive mucinous adenocarcinoma | + | + | - | WT | / |
| 1832 | F | 56 | N | I | Solid predominant | + | + | - | WT | / |
| 1837 | M | 56 | C | I | Solid predominant | + | + | - | WT | / |
| 1873 | M | 58 | C | I | Acinar predominant | + | + | - | WT | / |
| 1984 | F | 61 | N | I | Lepidic predominant | + | + | - | WT | / |
| 2008 | F | 62 | N | II | Solid predominant | + | + | - | WT | / |
| 2036 | M | 63 | N | I | Solid predominant | + | + | - | WT | / |
| 2067 | M | 63 | Former | III | Solid predominant | + | + | - | WT | / |
| 2083 | M | 65 | N | I | Micropapillary predominant | W+ | + | - | WT | + |
| 2108 | M | 65 | C | II | Papillary predominant | + | + | - | WT | / |
| 2131 | F | 66 | N | II | Papillary predominant | + | + | - | L858R | / |
| 2178 | F | 68 | N | III | Invasive mucinous adenocarcinoma | + | + | - | WT | / |
| 2207 | F | 70 | N | I | Invasive mucinous adenocarcinoma | + | + | - | / | / |
| 2272 | M | 75 | N | I | Solid predominant | + | + | - | WT | / |
| 2284 | F | 76 | N | I | Invasive mucinous adenocarcinoma | + | + | - | WT | / |

All specimens were formalin-fixed-paraffin-embedded (FFPE) from surgical resections. F, female; M, male; C, current; N, never; IHC, immunohistochemistry; FISH, fluorescence in-situ hybridization; 19DEL: deletion in 19 exon; L858R: L858R mutation in 21 exon; WT: wild type; W+: weak positivity
